# Robotic mechanical wounding is sufficient to induce phenylacetaldoxime accumulation in *Tococa quadrialata*

**DOI:** 10.1080/15592324.2024.2360298

**Published:** 2024-05-30

**Authors:** Kilian Lucas Ossetek, Andrea Teresa Müller, Axel Mithöfer

**Affiliations:** Research Group Plant Defense Physiology, Max Planck Institute for Chemical Ecology, Jena, Germany

**Keywords:** Phenylacetaldoxime, *Tococa quadrialata*, herbivory, wounding, MecWorm, SpitWorm

## Abstract

This study investigated the accumulation of phenlyacetaldoxime (PAOx) and PAOx-Glc in *Tococa quadrialata* leaves in response to herbivore infestation and mechanical wounding. Results show that PAOx levels peaked at 24 h post-infestation, while PAOx-Glc remained present for several days. The accumulation of PAOx began as early as 3 h after herbivory, with PAOx-Glc significantly increased after 6 h. Mechanical wounding induced similar responses in PAOx and PAOx-Glc accumulation as herbivory, suggesting that continuous tissue damage triggers the production of these compounds. Interestingly, SpitWorm-treated leaves showed the highest levels of both PAOx and PAOx-Glc, indicating that herbivore-derived oral secretions (OS) play a role in the induction of these compounds. Additionally, JA-independent PAOx production was found to be associated with tissue damage rather than specific known signaling compounds. Emission of benzyl cyanide and 2-phenylethanol, PAOx-derived plant volatiles, was observed in response to herbivory and SpitWorm treatment providing plant-derived OS, further highlighting the role of herbivore cues in plant defense responses.

## Introduction

Plants are sessile organisms that need to cope with the abiotic and biotic environment. As they are primary producers, they serve as a food source for countless organisms. To survive attacks from phytopathogens and herbivores, plants developed many different mechanisms to defend themselves. A classical way of plant defense against attackers is the production and accumulation of secondary or specialized metabolites, such as polyphenols, terpenoids, glucosinolates, alkaloids or cyanogenic glucosides, which contribute constitutively and inducibly to plant defense and can exhibit toxic or anti-nutritive effects on herbivorous insects and phytopathogenic microorganisms.^1,2^

**Figure 1.**
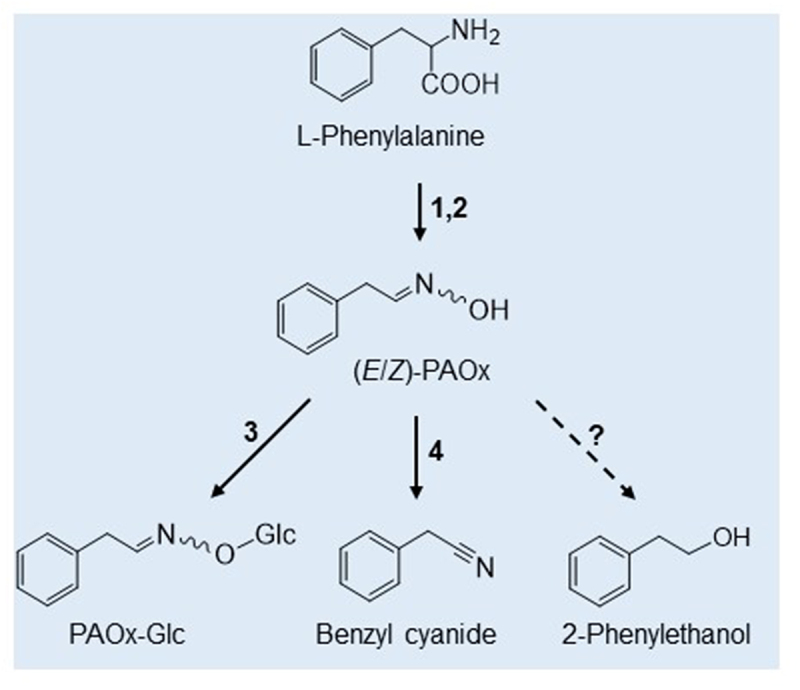
Schematic biosynthetic pathway of phenylacetaldoxime (PAOx) and related metabolites in *Tococa quadrialata*. Enzymes involved: 1,2 (CYP79A206, CYP79A207); 3 (UGT85A123); 4 (CYP71E76);? (unknown).

One widely distributed class of compounds that is related to plant defense is aldoximes, which often function as biosynthetic precursors for defense compounds or accumulate upon herbivory. This, for example, holds true for phenylacetaldoxime (PAOx), suggesting that PAOx itself is a defensive compound.^3,4^ PAOx is a known precursor for many active plant defense compounds such as the cyanogenic glucosides prunasin and amygdalin or the glucosinolate glucotropaeolin. These defense compounds are enzymatically degraded upon release through cell damage to produce hydrogen cyanide (cyanogenic glucosides) or isothiocyanate (glucosinolates), which can be toxic for herbivorous species.^5,6^ PAOx has been found as an intermediate or end product in a number of plants including *Populus trichocarpa*,^3^ *Zea mays*,^7^ *Erythroxylum fischeri* and *E. coca*,^8^ *Arabidopsis thaliana*,^9^ *Camellia sinensis*,^10^ *Prunus mume*,^11^ *Fallopia sachalinensis*^12^ and recently in *Tabernaemontana divaricata*, *Glycine max* and *Tococa quadrialata*.^4^ For the latter plant, the herbivore-induced biosynthesis of PAOx and the novel PAOx glucoside (PAOx-Glc) has just been elucidated, and PAOx-derived volatile defense compounds have been identified such as benzyl cyanide (BCN) and in a heterologous expression system 2-phenylethanol (Figure 1).^4^ While PAOx-Glc levels remain high for a few days after insect feeding, PAOx levels are high only during the feeding process, suggesting that the glucoside may represent the storage form of a defense-related compound. Strikingly, in *T. quadrialata* both PAOx and PAOx-Glc are inducible by herbivory but are restricted to the site of attack and cannot be detected systemically.^4^ Both compounds have also been found upon insect herbivory in *P. trichocarpa* and *T. divaricata* as well as upon elicitor treatment in *G. max* cell cultures.^4^ Thus, it seems justified to speculate that the formation and storage of PAOx and PAOx-Glc represents a widespread plant defense response.

Still, the nature of the trigger of PAOx induction remains an open question. Commonly, plants perceive threats through distinct highly conserved molecular patterns. These molecular patterns are also referred to as elicitors, signaling molecules or X-associated molecular patterns (XAMPs), where X represents the molecule’s origin. Elicitors that were found to induce plant defense and secondary metabolite production include bacterial flagellin protein, which originates from the bacterial flagella^13^ or fungal cell wall fragments, namely chitin- and chitosan-derived oligosaccharides.^14^ Both are considered pathogen-associated molecular patterns (PAMPs). In case of insect attacks, it is known that herbivory is a combination of mechanical wounding and herbivore-derived chemical signals.^15^ While the wounding process releases DAMPs (damage-associated molecular patterns), herbivore feeding in addition provides HAMPs (herbivory-associated molecular patterns). Examples of DAMPs are cellotriose and other fragments of cellulose or oligogalacturonides, which are fragments of pectin. These fragments emerge from the enzymatic degradation of the plant cell wall and serve as signaling molecules, which trigger plant defenses.^16–18^ HAMPs include larger proteins, small peptides, as well as fatty acids and derived conjugates or esters.^15^

Here, we aimed to analyze which treatment induces PAOx accumulation in *T. quadrialata*. Thus, to discriminate between sole wounding and real herbivory that includes the insect’s chemistry, we employed robotic caterpillars. First, we mimicked the mechanical feeding process of a feeding larva without the chemical components provided by the insect MecWorm;^19^ and second, we mimicked herbivory including the insect-derived chemistry SpitWorm.^20^ We further tried to elicit PAOx by treatments with different elicitors representing PAMPs and DAMPs. Our results suggest that continuous mechanical tissue damage is sufficient for the formation of PAOx and derivatives, but the herbivore’s chemistry has an additional impact.

## Materials and methods

### Plants and insects

Neotropical myrmecophytic *Tococa quadrialata* Naudin plants (recently renamed as *Miconia microphysca* Michelang. Melastomataceae) were grown from seeds in a glasshouse as described.^4^ Experiments were performed with mature plants (ca. 30–60 cm height). Generalist herbivore *Spodoptera littoralis* (Egyptian cotton leafworm; Lepidoptera, Noctuidae) larvae were hatched from eggs obtained from Syngenta Crop Protection AG (Switzerland) and reared as described.^19^ Second and third instar larvae were chosen for feeding experiments and starved 18–24 h prior to plant feeding. Before the collection of larval oral secretion (OS), fourth to fifth instar larvae fed on *T. quadrialata* leaves or artificial diet for 24 h. A pipette was used to collect OS that was released by the caterpillar after holding the larvae in place and irritating them with forceps. The OS obtained was stored at −20°C.

### Mechanical wounding and herbivory

For continuous mechanical wounding mimicking herbivory, MecWorm was employed.^19^ This mechanical device was set to wound the leaves in a defined area of a circle with a radius of 4 cm and 4 sec between each hit. This approach was complemented with leaves subjected to SpitWorm wounding. The SpitWorm enhances the abilities of the MecWorm by supplying larval OS to the wounding site.^20^ Here, the collected OS was centrifuged (10 min at room temperature 16,000 rcf) to remove larger particles and then diluted 1:10 in dH_2_O. A syringe pump was used to transfer the OS to the wounding site, using an uncoated capillary (inner diameter: 0.25 mm, outer diameter: 0.36 mm). The pump was adjusted to a flow rate of 600 nL min^−1^ and a syringe diameter of 0.416 mm. The capillary was fixed to the stamp of the MecWorm device, enabling a constant supply of OS to the wounding site. Untreated leaves were used as control. For herbivory, one 3rd instar *S. littoralis* larva was kept in a clip cage (diameter 3.6–3.9 cm) and allowed to feed in the given area. At the end of all treatments, a 1 cm ring was cut around the wounding sites excluding the directly wounded material. All leaves were harvested 24 h after the beginning of the particular experiments. All plant material was directly frozen in liquid nitrogen and stored at −80°C until further processing.

To investigate the temporal accumulation of PAOx and PAOx-Glc, between 5 and 20 3rd to 4th instar *S. littoralis* larvae were placed on a *T. quadrialata* leaf and kept there using a plastic bag. The larvae could feed for the indicated time (0, 1, 3, 6, 14, 24 h) before harvesting the leaves. All collected plant material was frozen in liquid nitrogen and stored at −80°C until further processing.

### Chemical analyses

For the analysis of volatiles emitted from *T. quadrialata* plants that were under treatment with either MecWorm, SpitWorm, or *S. littoralis*, a push-pull system was used according to Müller et al.^4^ Briefly, during treatments, charcoal-filtered air was pushed into a closed chamber or PET-bag (flow rate of 0.5 L min^−1^) and pulled through a PoroPak (Alltech, FL, USA) filter (flow rate of 0.3 L min^−1^). After 24 h, the absorbed volatiles were eluted from the filter using two times 100 μL of dichloromethane (DCM, Carl Roth GmbH + Co. KG) containing 10 ng μL^−1^ n-bromodecane (Sigma-Aldrich, Taufkirchen, Germany) as an internal standard. Volatiles were analyzed by GC-MS and quantified by GC-FID as described.^21^ Compounds were identified by comparison to the authentic standards. Non-volatile compounds such as PAOx, PAOx-Glc, and the defense-related phytohormone jasmonic acid isoleucine conjugate (JA-Ile) were extracted from frozen ground plant tissue, analyzed with LC-MS/MS and identified as described in detail before.^4^

### Elicitor treatments

Elicitors and other compounds were obtained from different suppliers: chitosan (Sigma-Aldrich, Taufkirchen, Germany), cellotriose (Megazyme, Bray, Ireland), oligogalacturonides (DP = 10–15) (Elicityl S.A., Crolles, France), flagellin 22 (EZbiolabs, Carmel, IN, USA), ß-glucan (raw elicitor fraction isolated from cell walls of *Phytophthora sojae*^22^) and PAOx (synthesized according to Müller et al.^4^). Leaves of *T. quadrialata* were washed in distilled water and carefully wiped off using paper towels. For each treatment, leaves from six different plants were used, taking one leaf from each plant. Leaf disks of 2 cm diameter were stamped out of each leaf using a stencil. All leaf disks of one treatment were syringe vacuum infiltrated at once while preserving the order of the leaf disks. After the disks were fully infiltrated (4× by applying positive and negative pressure in the syringe), they were again wiped off using paper towels. All leaf disks of one treatment were filled into a closed glass container equipped with a wet paper that kept humidity high to reduce and slow down any drying. After 5 h, the leaf disks were weighted, flash frozen in liquid nitrogen and extracted with methanol for the analysis of PAOx, PAOx-Glc and JA-Ile as described.^4^

### Statistics and data visualization

All statistical analyses were performed in R (version 4.3.1, R Core Team 2022) within R Studio environment^23^ or Sigma Plot (version 14.0, Systat Software, Inc.). Statistical assumptions for one-way ANOVA (normal distribution and homoscedasticity of the residuals) were confirmed by diagnostic plots.

Data visualization was accomplished using R and R-Studio as mentioned. All bar plots show the mean ± standard error of the mean (SEM) for the respective treatment. Alterations are mentioned in the plot description. All box plots shown are built upon the following structure: center line, median; box limits, upper and lower quartiles; whiskers, 1.5× interquartile range; orange dot, mean).

## Results

**Figure 2.**
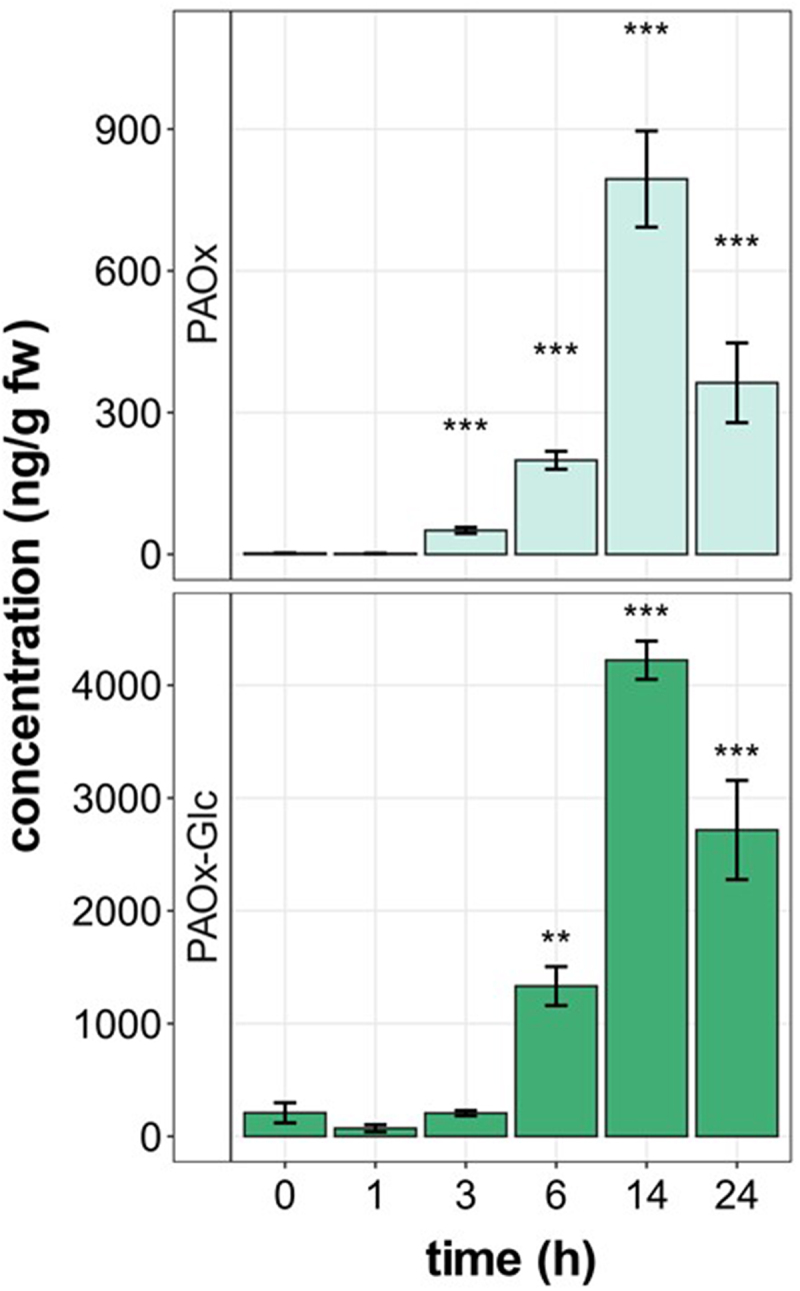
PAOx and PAOx-Glc concentrations in *Tococa quadrialata* leaves determined after *Spodoptera littoralis* larvae feeding for the indicated time. Significant differences to the respective control (*t* = 0 h) are marked by asterisks (** *p* < .01***, *p* < .001) and were determined by one-way-ANOVA on ln-transformed data (F_PAOx_ = 70.44, p_PAOx_ <0.001; F_PAOx – Glc_ = 16.99, *p* < .001; *n* = 3–4) and Holm-Sidak post hoc test.

In a recent study, phenlyacetaldoxime (PAOx) and its glucoside (PAOx-Glc) were detected after herbivore infestation in *T. quadrialata* leaves. Induced PAOx levels were reported to peak at 24 h and diminish thereafter, while the glucoside remained present for several days.^4^ Liao et al.^10^ reported accumulation of PAOx in tea already within 6 h of mechanical wounding, but when PAOx and PAOx-Glc start to accumulate in the plant tissue after herbivory, their accumulation time remains unknown. In order to analyze earlier responses, the feeding experiment was conducted again on *T. quadrialata* leaves, employing *S. littoralis*. Larvae were allowed to feed for different periods up to 24 h, and the leaves were collected at different time points (Figure 2). PAOx and PAOx-Glc concentrations were analyzed in the harvested leaves. The first significant accumulation of PAOx could be detected after 3 h of herbivory, while the glucoside was significantly increased after 6 h of herbivory. Both compounds showed still high concentrations up to 1 d of feeding. Therefore, the following treatments were conducted for 24 h before leaves were harvested.

**Figure 3.**
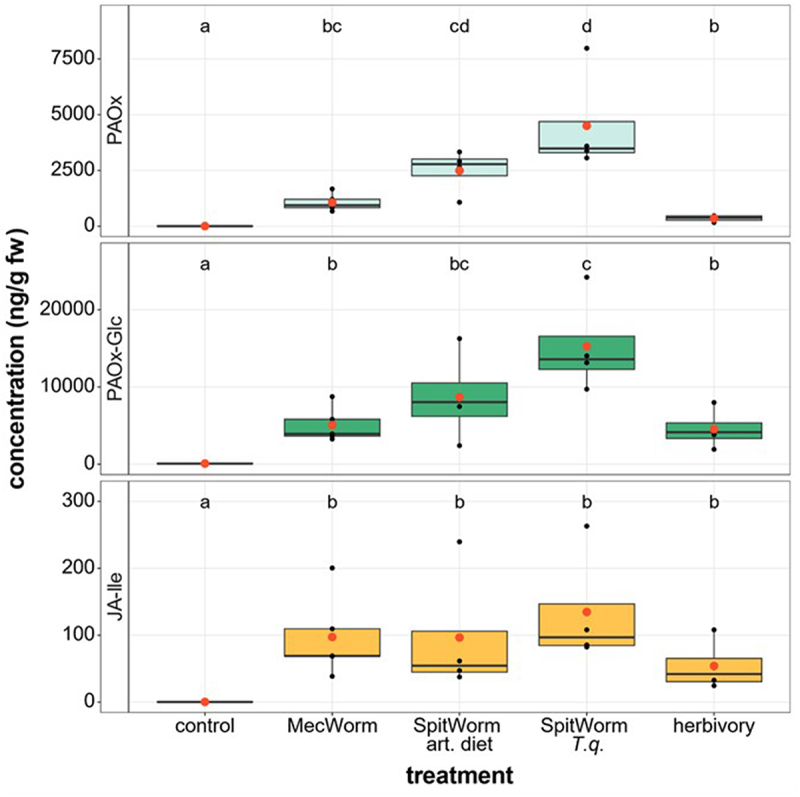
PAOx, PAOx-Glc, and JA-Ile determination in *T. quadrialata* leaves upon treatments with mechanical wounding (MecWorm), mechanical wounding and OS (SpitWorm), or *Spodoptera littoralis* larvae (herbivory) for 24 h. OS was collected from larvae that fed on *T. quadrialata* (*T.Q*.) or on artificial diet (art. diet). Statistically significant differences between groups are marked by different letters (*p* < .05) and were determined by one-way-ANOVA on ln-transformed data (F_PAOx_ = 157, p_PAOx_ <0.001; F_PAOx – Glc_ = 61.7, p_PAOx – Glc_ <0.001; F_JA – Ile_ = 61.76, p_JA – Ile_ = 0.001, *n* = 4–5) and TukeyHSD post hoc test.

To learn more about what triggers the accumulation of these compounds, mechanical wounding that mimicked insect feeding was performed and compared with real insect herbivory. As expected, compared to the control, the bioactive JA-Ile accumulated to the same significantly higher levels in plants treated with either MecWorm, SpitWorm, or *S. littoralis*, respectively, indicating that all plants responded to the treatments (Figure 3). Although MecWorm treatment caused higher accumulation of PAOx and PAOx-Glc, the difference to herbivory was not statistically significant. However, the findings of these experiments suggested that continuous wounding is sufficient for the induction of PAOx and PAOx-Glc. Additionally, the influence of herbivore-derived OS on PAOx and PAOx-Glc induction was investigated by damaging leaves with the SpitWorm, which supplies a constant flow of oral secretion to the wounding site. Levels of PAOx analyzed in the sampled leaf tissue were the lowest in the positive control herbivory and the significantly highest in SpitWorm-treated leaves. For PAOx-Glc, the highest amount also accumulated in SpitWorm-treated leaves, while the treatments MecWorm and herbivory showed similar levels of the compound (Figure 3). Strikingly, we also found a slight difference in the accumulation of PAOx and PAOx-Glc depending on the origin of the OS. When the larvae were fed with artificial food, the response of the plants to SpitWorm was less pronounced than when the larvae fed on *T. quadrialata* leaves (Figure 3).

**Figure 4.**
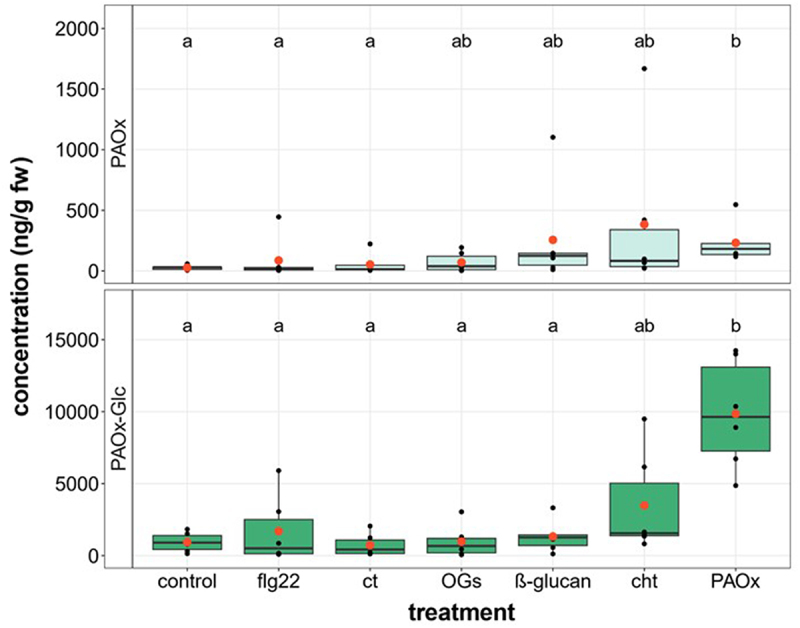
PAOx and PAOx-Glc determination in *T. quadrialata* leaves upon treatment with various elicitors. Treatments: control = water; flg22 = flagellin 22 (10 µM); ct = cellotriose (10 µM); OGs = oligogalacturonides (10 µM); 1,3–1,6-ß-glucan (0.5 mg ml^−1^); cht = chitosan (10 µM); PAOx (1 mM). Different letters display statistical differences between groups (*p* < .05) and were determined by the comparison of a linear mixed-effects model with a baseline model to determine the effect of the applied treatments (L-ratio_PAOx_ = 19.78, p_PAOx_ <0.01; L-ratio_PAOx – Glc_ = 30.67, p_PAOx – Glc_ <0.001; *n* = 6) and Tukey Contrasts post hoc test with Holm correction.

PAOx and PAOx-Glc induction was found to be JA-independent in *T. quadrialata*,^4^ but rather associated with tissue damage. To investigate which cues might elicit PAOx production, various elicitors were infiltrated into *T. quadrialata* leaf tissue. PAOx and PAOx-Glc concentrations were analyzed in the infiltrated leaf material, which was harvested 5 h after the infiltration. In general, high concentrations were chosen for each signaling compound (XAMP), to safely elicit a reaction. In addition to the bacterial flagellum protein ’flagellin22’ (flg22) as a bacterial PAMP, chitosan (cht) as a PAMP derived from the fungal cell wall as well as the herbivore exoskeleton, ß-glucan as an oomycete-derived PAMP, cellotriose (ct) and a mix of oligogalacturonides (OGs) were tested as they represent typical DAMPs that are released upon tissue wounding. None of the elicitors tested induced the production of PAOx or PAOx-Glc. PAOx-Glc was only elevated in leaves infiltrated with the positive control PAOx (Figure 4). In addition, JA-Ile was analyzed to see if the infiltration process induced wounding stress. Indeed, the JA-Ile concentration in the control plants was detected in the same range as known for pattern wheel-treated leaves (7.5 and 5 ng g^−1^ fw, respectively),^4^ while in non-treated leaves, JA-Ile concentration was indicated with 2 ng g^−1^ fw.^4^ Concerning the elicitor treatments, compared to the control, no significant differences were detected in any of the treatments.

**Figure 5.**
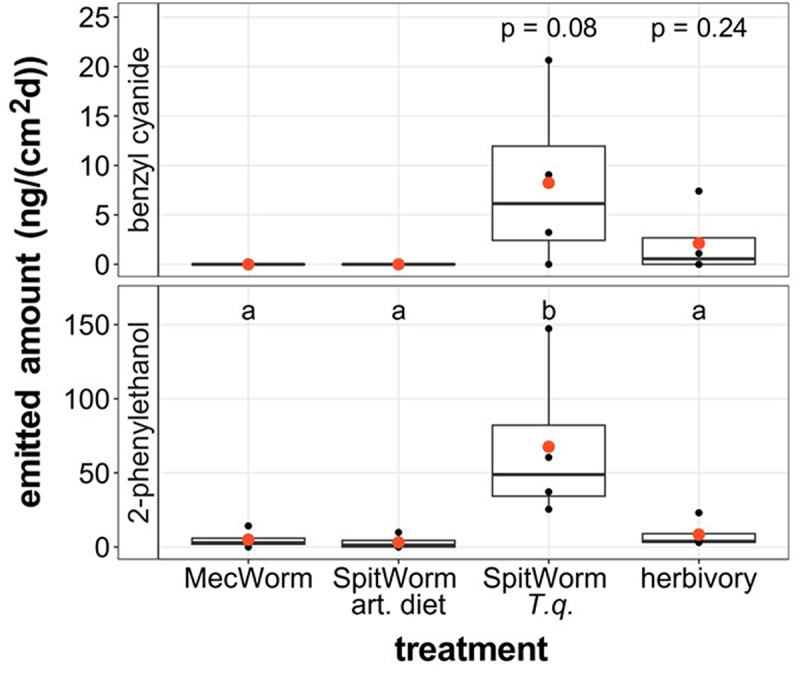
Volatile (2-phenylethanol, PEA; benzyl cyanide, BCN) determination in *T. quadrialata* leaves upon treatments with mechanical wounding (MecWorm), mechanical wounding and OS (SpitWorm), or *Spodoptera littoralis* larvae (herbivory) for 24 h. OS was collected from larvae that fed on *T. quadrialata* (*T.Q*.) or on an artificial diet (art. diet). Statistically significant differences between groups are marked by different letters (*p* < .05) and were determined by one-way-ANOVA on ln(x + 1)-transformed data (F_PEA_ = 3.803, p_PEA_ <.05; *n* = 4) and TukeyHSD post hoc test. For BCN differences, statistical differences were determined by Student’s t-tests against 0 with ln(x + 1)-transformed data (t_Spitworm_ = 2.587, p_Spitworm_ = 0.08, t_Herbivory_ = 1.431, p_Herbivory_ = 0.24; µ = 0; *n* = 4).

Insect feeding leads to the emission of PAOx-derived nitrile benzyl cyanide in *T. quadrialata*.^4^ In the experiment with MecWorm and SpitWorm, the headspaces of *T. quadrialata* leaves were also collected and analyzed for emitted PAOx-derived plant volatiles. The emission of benzyl cyanide was slightly induced by *S. littoralis* herbivory and SpitWorm providing *T. quadrialata* OS. MecWorm-treated leaves emitted no benzyl cyanide. Benzyl cyanide was detectable only in Spitworm and herbivory treated leaves, however there was no statistical significance between any of the treatments. In addition, all three treatments led to the emission of very small quantities of 2-phenylethanol from the leaf (Figure 5). Only leaves treated with the SpitWorm providing *T. quadrialata* derived OS (*T.q*.-OS) emitted significantly higher amounts of 2-phenylethanol than leaves treated with MecWorm, herbivory, or SpitWorm providing artificial diet OS (art.diet-OS) (Figure 5).

## Discussion

Detecting biotic stresses is crucial for the plant’s survival. The ability to respond to environmental cues allows plants to react accordingly by, for example, inducing secondary or specialized metabolite upon demand. This enables the plant to use resources efficiently, which circumvents the risk of plant growth reduction and developmental setbacks.^1,2,24^ Pathogen infections or herbivore infestations are often the triggers for the induction of defense-relevant metabolites. During the attack, signaling compounds (XAMPs) from various origins are involved in the recognition process. However, these compounds are often not known.

PAOx is a plant-derived oxime found in plant species from various plant families, among others in *P. trichocarpa*,^3^ *Z. mays*,^7^ *C. sinensis*,^10^ the myrmecophyte *T. quadrialata*^4^ and many more. Additionally, all analyzed angiosperms possess the genetic ability to produce oximes.^25^ Previous studies have shown that PAOx and similar plant-derived oximes are key intermediates for multiple classes of secondary metabolites, which play important roles in plant defense and ecological interactions .^26–28^ PAOx accumulates in larger quantities in some plants, and its newly discovered glucoside accumulates in even higher amounts in *T. quadrialata* (Müller et al. 2024),^4^ suggesting that PAOx and PAOx-Glc contribute to the plant’s defense under herbivore attack. Therefore, this study aims to find out which particular part of the insects’ feeding process is necessary for the induction of PAOx and PAOx-Glc in *T. quadrialata*.

Knowing that within 24 h of insect feeding PAOx and PAOx-Glc strongly accumulated in *T. quadrialata* leaves (Figure 2), we used robotic tools that can mimic insect herbivory (MecWorm^19^ and SpitWorm^20^) in comparison to *S. littoralis* feeding. While MecWorm treatment is similar to the sole mechanical wounding process and releases DAMPs, SpitWorm in addition provides insect-derived OS and, thus, HAMPs. The results show that mechanical wounding is sufficient to induce both PAOx and PAOx-Glc (Figure 3). However, the presence of OS further increased the amount of both compounds (Figure 3). Here, the origin of the OS seems to be of further importance, as PAOx and PAOs-Glc accumulation is even higher when the OS-providing insects were previously fed with *T. quadrialata* leaves instead of artificial diet. Two scenarios can explain this finding. First, the OS contains DAMPs that originate from the leaves and were taken up by the feeding larva. Second, plant metabolites were taken up, modified in the insect gut and became HAMPs. This latter scenario is known, e.g., for inceptins.^29,30^ Inceptins are peptides derived from the chloroplastic ATP synthase γ-subunit but processed to an active form of the elicitor in the insect gut. In both cases, the signaling compounds are again presented to the wounding site. In the first scenario, the DAMP concentrations increase, in the second scenario the HAMPs contribute to the reactions induced by DAMPs.

Differences in PAOx concentrations could also have been a result of PAOx-derived volatile emission. However, the data show that there is no clear correlation between volatile emission and the accumulated amount of PAOx (Figures 3, 5). Upon SpitWorm with *T.q*.-OS treatment, the highest amount of BCN and 2-phenylethanol was emitted but almost nothing upon SpitWorm with art.diet-OS or MecWorm although PAOx levels were high (Figures 3, 5). In addition to the emission of the PAOx-derived BCN, 2-phenylethanol was emitted in larger quantities upon all treatments. This compound was not described for *T. quadrialata* before, although heterologous expression of the biosynthetic genes for PAOx synthesis, CYP79A206 and CYP79A207, and their activities suggested the presence of 2-phenylethanol.^4^ Both volatiles might be involved in defense and are emitted under attack from the leaf tissue in *P. trichocarpa* and *T. quadrialata*.^3,4^ Alcohol 2-phenylethanol was shown to be important in plant–predator interactions.^27^

It is reasonable to suggest that PAOx induction is further mediated by JA signaling, as in many other defense-related compounds.^1^ However, the empirical data is mixed. A JA-dependent induction of PAOx was shown in *E. fischeri* and *E. coca* (JA-induced)^8^ as well as in *F. sachalinensis* (MeJA-induced).^12^ In contrast, results in *T. quadrialata* suggest JA-independent, continuous mechanical wounding as the main trigger of PAOx induction.^4^ For *T. quadrialata*, this evidence is also supported by our results, as all treatments induced JA-Ile to the same extent, but PAOx and PAX-Glc to different levels (Figure 3).

Previous studies reported multiple biological stimuli as inducers of PAOx induction in various plant species.^3,3,4,10,12,31^ These include herbivory, mechanical wounding as well as plant pathogens. All these stresses provide a plethora of possible elicitors. Therefore, we studied if a collection of known and commonly used DAMPs and PAMPs may induce PAOx and PAOx-Glc accumulation by infiltration into *T. quadrialata* leaf disks. None of the tested elicitors caused the induction of PAOx and PAOx-Glc, although the positive control with PAOx showed that the methods of infiltration worked well (Figure 4).

The data of this study suggest that the induction of PAOx and PAOx-Glc and their derivatives is associated with continuous wounding. These results add to the findings in *T. quadrialata* reported by Müller et al.^4^ and are similar to the pattern found in *C. sinensis* by Liao et al.^10^ It should also be emphasized at this point that even in myrmecophytic plants such as *T. quadrialata*, which are protected by mutualistic ants, PAOx metabolism is still induced.^21^ This suggests that this oxime might be involved very likely in response to tissue damage rather than infestation. However, the wounding-released DAMPs and/or the putative insect-derived HAMPs that are involved still remain to be identified.

## Data Availability

Data will be made available upon reasonable request to the corresponding author.
